# Analysis of *Canis *mitochondrial DNA demonstrates high concordance between the control region and ATPase genes

**DOI:** 10.1186/1471-2148-10-215

**Published:** 2010-07-16

**Authors:** Linda Y Rutledge, Brent R Patterson, Bradley N White

**Affiliations:** 1 Environmental and Life Sciences Graduate Program, DNA Building, Trent University, 2140 East Bank Drive, Peterborough, ON, K9J 7B8, Canada; 2 Ontario Ministry of Natural Resources, Wildlife Research Development Section, Trent University, DNA Building, 2140 East Bank Drive, Peterborough, ON, K9J 7B8, Canada; 3 Biology Department, Natural Resources DNA Profiling & Forensic Centre, DNA Building, Trent University, 2140 East Bank Drive, Peterborough, ON, K9J 7B8, Canada

## Abstract

**Background:**

Phylogenetic studies of wild *Canis *species have relied heavily on the mitochondrial DNA control region (mtDNA CR) to infer species relationships and evolutionary lineages. Previous analyses of the CR provided evidence for a North American evolved eastern wolf (*C. lycaon*), that is more closely related to red wolves (*C. rufus*) and coyotes (*C. latrans*) than grey wolves (*C. lupus*). Eastern wolf origins, however, continue to be questioned. Therefore, we analyzed mtDNA from 89 wolves and coyotes across North America and Eurasia at 347 base pairs (bp) of the CR and 1067 bp that included the ATPase6 and ATPase8 genes. Phylogenies and divergence estimates were used to clarify the evolutionary history of eastern wolves, and regional comparisons of nonsynonomous to synonomous substitutions (*d*N/*d*S) at the ATPase6 and ATPase8 genes were used to elucidate the potential role of selection in shaping mtDNA geographic distribution.

**Results:**

We found high concordance across analyses between the mtDNA regions studied. Both had a high percentage of variable sites (CR = 14.6%; ATP = 9.7%) and both phylogenies clustered eastern wolf haplotypes monophyletically within a North American evolved lineage apart from coyotes. Divergence estimates suggest the putative red wolf sequence is more closely related to coyotes (D_xy_CR = 0.01982 ± 0.00494 SD; D_xy_ATP = 0.00332 ± 0.00097 SD) than the eastern wolf sequences (D_xy_CR = 0.03047 ± 0.00664 SD; D_xy_ATP = 0.00931 ± 0.00205 SD). Neutrality tests on both genes were indicative of the population expansion of coyotes across eastern North America, and *d*N/*d*S ratios suggest a possible role for purifying selection in the evolution of North American lineages. *d*N/*d*S ratios were higher in European evolved lineages from northern climates compared to North American evolved lineages from temperate regions, but these differences were not statistically significant.

**Conclusions:**

These results demonstrate high concordance between coding and non-coding regions of mtDNA, and provide further evidence that the eastern wolf possessed distinct mtDNA lineages prior to recent coyote introgression. Purifying selection may have influenced North American evolved *Canis *lineages, but detection of adaptive selection in response to climate is limited by the power of current statistical tests. Increased sampling and development of alternative analytical tools will be necessary to disentangle demographic history from processes of natural selection.

## Background

Mitochondrial DNA (mtDNA) has been widely used in phylogenetic studies aimed at answering questions related to ecology and evolution. Its maternal inheritance, lack of recombination, high copy number, variable substitution rates across regions, high mutation rate compared to nuclear DNA, and role in energy production [1] make it an attractive genome for research that aims to understand species relationships, evolutionary history, and demographic patterns within both contemporary and historic contexts. The control region of the mitochondria can be particularly useful in understanding genetic relationships of recently diverged species because it contains hypervariable regions [2]. The high variation can, however, be problematic for inferring phylogenetic relationships due to mutation rate heterogeneity among nucleotide sites [3] and high rates of homoplasy [4,5] that can lead to ambiguous phylogeographic patterns [6]. Although not without its own peculiarities [1], coding regions of the mtDNA genome may help clarify genetic and spatial relationships of species inferred from the control region alone. Although all regions of mtDNA are linked and the entire mtDNA genome is inherited as a single molecule without recombination, coding and non-coding sections exhibit different mutation rates due to higher selective forces acting on genes that code for functional proteins [1]. Thus, different patterns of diversity, divergence, and phylogenetic clustering may be evident when comparing regions under divergent selective forces.

In addition to complementing control region phylogenies, analysis of mtDNA coding regions may help resolve geographical distribution patterns because coding regions of the mtDNA are under strong selection due to their fundamental role in energy and heat production [7,8]. There is growing evidence that purifying selection on mtDNA coding regions has been important in shaping the evolution and distribution of mtDNA [8-11]. Additionally, adaptive selection may be important [12] with climatic adaptation acting as an influential factor in mtDNA geographic distribution [13-15], although some have disputed the climate hypothesis [8,16,17]. Despite this controversy, most agree that the evolution of mtDNA is likely more complex than any single factor could account for. Recent research, however, suggests that the mtDNA ATPase genes in particular, may be influenced by positive selection [8].

To date, studies of North American *Canis *phylogenetics have relied heavily on the mtDNA control region to infer species relationships and evolutionary history [18-27]. Phylogenetic analysis of the control region provided initial evidence for a North American-evolved wolf, the eastern wolf (*Canis lycaon*), that shared an evolutionary history with red wolves (*C. rufus*) and coyotes (*C. latrans*) independent of grey wolves (*C. lupus*) that evolved in Eurasia and dispersed into North American approximately 300,000 years ago [19,20]. Since then, various research has added to the growing evidence supporting the eastern wolf as a distinct species [28], including genetic analysis of historic [20] and ancient [25] samples. Despite this, the lineage of the eastern wolf continues to be challenged, in part because evidence from coding regions that are under selection is lacking [29]. Indeed, phylogenetic research on wild canids has rarely ventured beyond the mtDNA control region.

Phylogenetic analysis of nuclear *Canis *markers is complicated by historic and recent hybridization between coyotes, eastern wolves, and gray wolves [24,25,27]. The advantage of studying a circular, non-recombining marker like mtDNA is that patterns of evolutionarily independent lineages can be more easily identified under complicated demographic histories. Here, we use both the control region and the ATPase coding region of the mtDNA genome to infer phylogenetic relationships of the eastern wolf to other *Canis *species. High levels of hybridization and probable incomplete lineage sorting in eastern North American *Canis *species [24,25,27] also complicate species inferences from phylogenetic analysis of mtDNA. We, therefore, focus on divergence of phylogenetic clades to provide an understanding of the evolutionary relationships and the role that selection has played in the geographic distribution of *Canis *mtDNA haplotypes. Our study is the first to provide a substantial analysis of the mtDNA ATPase region in wild *Canis *populations. For clarity, a list of abbreviations is included at the end of the manuscript.

## Results & Discussion

### Diversity and phylogenetic analysis

After combining sequences obtained from our study with 9 from Genbank we analyzed 83 control region sequences (347 bp; h = 29) and 89 ATPase sequences (1067 bp; h = 26) (Table 1). Genbank accession numbers for sequences generated in this study are HM755678-HM755718. Similar to other studies on the control region [18], we found a higher proportion of NW coyote clustering haplotypes per sample size at both the control and ATPase region (0.38, 0.33) compared to OW haplotypes (0.29, 0.26). (Haplotype assignments to specific samples are shown in Additional File 1: Summary of sample locations and mtDNA control region and ATPase region haplotypes). Overall, we found high concordance between results from the control region and those from the ATPase region despite the different selective forces acting on the two regions. The control region had high variability but only 1.5 times more variable sites and 1.6 times higher nucleotide diversity per site (Pi) compared to the ATPase region (Table 1). Within specific phylogenetic clades, however, Pi was noticeably higher in the control region than the ATPase region (Table 1). The higher diversity in the control region is expected since it is not known to code for functional proteins [30] and has been identified as a mutational hot spot [2]. Recent work, however, suggests that mutational hotspots also occur in coding regions of the mtDNA [31] making the high variation reported here for ATPase genes somewhat less surprising. There were fewer ATPase8 than ATPase6 haplotypes, due to the shorter sequence length of ATPase8, and grey wolves from Sweden, Russian, Spain, and Canada all shared an ATPase8 haplotype (Additional File 1), suggesting that ATPase8 is a highly conserved gene region.

**Table 1:** Polymorphism within the mtDNA control region (CR) and ATPase regions

Clade	Number of sites analyzed	Sample size (n)	Number of haplotypes (h)	Number of variable sites (%)	Nucleotide diversity (Pi) per site	Pi SD
**CR**						
All_CR_	335	83	29	49 (14.6)	0.04257	0.00214
NW_CRall_	337	56	20	30 (8.9)	0.01718	0.00223
NW_CR1ew_	341	9	2	4 (1.2)	0.01173	0.00587
NW_CR2coyI+CR3coyII _	337	47	18	25 (7.4)	0.01424	0.00161
NW_CR2coyI_	338	16	7	15 (4.4)	0.0155	0.00259
NW_CR3coyII_	339	31	11	14 (4.1)	0.01019	0.0016
OW_CRall_	345	24	7	11 (3.2)	0.01408	0.00176
OW_CR4gwNA_	345	20	3	2 (0.6)	0.00386	0.00129
OW_CR5gwEU_	345	4	4	7 (2.0)	0.01208	0.00227
**ATPase**						
All_ATP_	1067	89	26	104 (9.7)	0.02596	0.00177
NW_ATPall_	1067	61	18	42 (3.9)	0.00543	0.00076
NW_ATP1ew_	1067	12	2	5 (0.5)	0.00469	0.00234
NW_ATP2coyI+ATP3coyII _	1067	49	16	33 (3.1)	0.00441	0.00056
NW_ATP2coyI_	1067	37	10	16 (1.5)	0.003	0.00059
NW_ATP3coyII_	1067	12	6	17 (1.6)	0.00594	0.00073
OW_ATPall_	1067	27	7	19 (1.8)	0.00643	0.00157
OW_ATP4gwNA_	1067	22	2	1 (0.09)	0.00094	0.00047
OW_ATP4gwEU_	1067	5	5	17 (1.6)	0.00731	0.00164
**ATPase6**						
All_ATP_	681	89	23	65 (9.5)	0.02513	0.00016
NW_ATPall_	681	61	16	28 (4.1)	0.00592	0.00079
NW_ATP1ew_	681	12	2	5 (0.7)	0.00734	0.00367
NW_ATP2coyI+ATP3coyII _	681	49	14	22 (3.2)	0.00495	0.00064
OW_ATPall_	681	27	7	16 (2.3)	0.00839	0.00133
OW_ATP4gwNA_	681	22	2	1 (0.1)	0.00147	0.00073
OW_ATP4gwEU_	681	5	5	14 (2.1)	0.00881	0.00142
**ATPase8**						
All_ATP_	204	89	14	23 (11.3)	0.03507	0.00629
NW_ATPall_	204	61	10	10 (4.9)	0.0111	0.00143
OW_ATPall_	204	27	4	4 (2.0)	0.01062	0.00311

Analysis was conducted in DnaSP v. 5.10 with nucleotide diversity calculated according to [60]. Gaps were excluded in the analysis. The full 1067 bp of the ATPase region is section 7729 - 8795 in the *Canis *complete mtDNA genome (Genbank Accession DQ480510) and includes gene regions ATPase8 (204 bp), ATPase6 (681 bp), plus flanking regions that overlap with COX2 and COX3 genes. Subscripts refer to clade designations in Figures 2a and 2b. CR = control region; SD=standard deviation.

**Figure 1:**
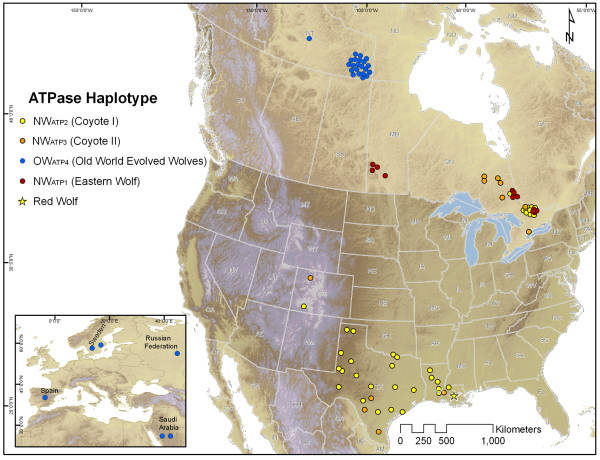
**Map of ATPase haplotype distribution**. Map of sample locations (circles) showing ATPase haplotype distribution. Colours represent different major clades indicated in Figures 2a and 2b. Specific locations for Genbank samples from Eurasia, Colorado, and the solitary sample shown in the Northwest Territories were unavailable so a random location within the country or state of origin was chosen.

**Figure 2:**
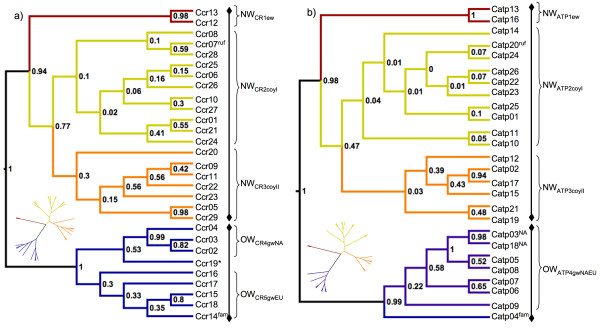
**Cladograms of *Canis *mtDNA CR and ATPase sequences**. Cladograms of *Canis *sequences from Bayesian analysis in BEAST of a) 347 bp of the mitochondrial DNA (mtDNA) control region and b) 1067 bp from the mtDNA ATPase region. ^fam ^is a Husky dog sample from Sweden; * is a wolf from Saudi Arabia; ^ruf ^is the red wolf sequence, NW represents New World evolved clades, OW represents Old World evolved clades, ^NA ^in b) identifies North American grey wolf samples. Node labels show posterior probabilities rounded to the nearest hundredth. For each genetic region the eastern wolf clade is shown in red (NW_CR1ew _& NW_ATP1ew_), coyote clades I and II are shown in yellow (NW_CR2coyI_, NW_ATP2coyI_) and orange (NW_CR3coyII _and NW_ATP3coyII_), and Old World (OW) clades from North America (OWCR4_gwNA_) and Eurasia (OWCR5_gwEU_, OWATP4_gwNAEU_) are shown in blue. Insets show radial view of tree.

There was a wide geographic distribution of NW coyote-like ATPase haplotypes (Figure 1). Cladograms from each genetic region had very similar topologies, which is indicative of the haplotype association of the linked regions. Eastern wolf sequences (Ccr13, Ccr12; Catp13, Catp16) clustered monophyletically with high (> 0.9) posterior probability within the NW clade, but apart from coyotes, whereas the putative red wolf sequence clustered among coyote sequences within the NW_CR2coyI_/NW_ATP2coyI _clade (Figures 2a, b). Similar clustering of the red wolf control region sequence among coyote sequences has been reported [22], but the haplotype is attributed to red wolves because it is not known to occur in non-hybridizing coyotes from western North America. A similar argument has been made for coyote-like eastern wolf haplotypes [27,32]. These geographic distinctions of coyote-like sequences in eastern and red wolves combined with evidence of coyote-like sequences in eastern wolves prior to European settlement in North America [25] provide evidence for incomplete lineage sorting within the NW lineages, although ancient (~11,000 years ago) hybridization during the Wisconsin glaciation is difficult to rule out. This, combined with extensive hybridization in eastern *Canis *populations [23,24,26,27,33,34], makes species designations of wild *Canis *difficult when based on phylogenetic inference from mtDNA alone. For example, a conspecific nature of eastern wolves and red wolves is suggested by nuclear data [19], but is not demonstrated by analysis of mtDNA. These differences are not unexpected and do not undermine the conspecific nature of eastern wolves and red wolves because gene trees based on mtDNA are not necessarily indicative of specific species relationships, and discordance is often found when comparing mtDNA and nuclear genetic signatures [35]. Given the recent divergence of NW lineages (see below) these issues are not unexpected [35]. New approaches to phylogenetic analysis that utilize multiple loci, including nuclear genes, may help reconcile inferred *Canis *species relationships [35,36], although extensive hybridization will likely continue to plague contemporary species designations based on nuclear markers. Regardless, the distinction of the two eastern wolf haplotypes shown in both the control region and ATPase region is indisputable, providing clear evidence for the presence of a North American evolved wolf lineage, distinct from coyotes and grey wolves.

### Divergence and TMRCA

**Figure 3:**
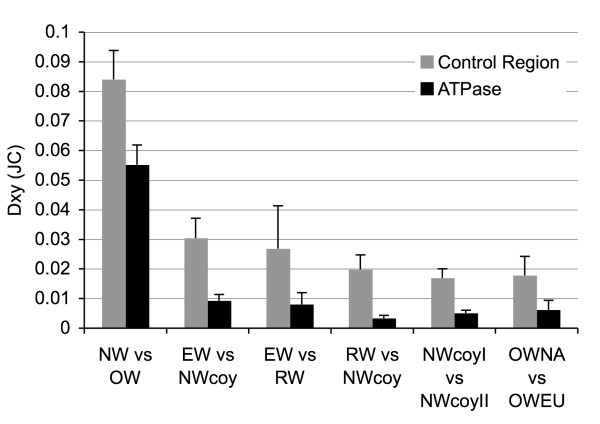
**Divergence between populations**. Divergence estimates between phylogenetic clades and putative species sequences at the mtDNA control region (347 bp) and ATPase region (1067 bp). Dxy (JC) = average number of nucleotide substitutions per site between populations (with Jukes and Cantor correction). Error bars are the standard deviation. NW = New World evolved sequences; OW = Old World evolved sequences, EW = eastern wolf sequences; RW = red wolf sequence; NWcoy = NWcoyI + NWcoyII; NWcoyI and NWcoyII represent the two coyote clades shown in Figures 2a and 2b; OWNA = Old World sequences from North America; OWEU = Old World sequences from Eurasia. Gaps were excluded in the analysis.

Fixed differences occurred in all but one comparison between groups (NW_ATP2coyI _vs. NW_ATP3coyII_) (Table 2). Whereas comparisons of the percentage of nucleotide differences were similar at both mtDNA regions when comparing deep divergences (ie. NW vs. OW), differences were substantially lower for more recent divergence comparisons within NW or OW evolved sequences (Table 2), indicative of possible homoplasy in the hypervariable sections of the control region [4,5]. Overall patterns of divergence (D_xyJC_) were similar for the control and ATPase regions, but estimates were consistently lower for ATPase compared to the control region (Figure 3), reflecting the lower observed mutation rate in ATPase.

**Table 2:** Nucleotide differences between clades and putative species

Comparison (Number of haplotypes)	Number of sites compared	Number of fixed differences	Average number of nucleotide differences between groups (%)
**CR**			
NW_CRall _(h = 20) vs OW_CRall _(h = 9)	335	14	26.6 (7.9)
NW_CR1(ew) _(h = 2) vs NW_CR2coyI+CR3coyII _(h = 18)	337	3	10.1 (3.0)
NW_CR1ew _(h = 2) vs RW (h = 1)	341	7	9 (2.6)
RW (h = 1) vs NW_CR2coyI+CR3coyII _(h = 17)	337	2	6.6 (2.0)
NW_CR2coyI _(h = 11) vs NW_CR3coyII _(h = 7)	337	1	5.6 (1.7)
OW_CR4gwNA _(h = 3) vs OW_CR5gwEU _(h = 4)	345	3	6.1 (1.8)
**ATPase**			
NW_ATPall _(h = 18) vs OW_ATPall _(h = 7)	1067	42	56.8 (5.3)
NW_ATP1ew _(h = 2) vs NW_CR2coyI+CR3coyII _(h = 16)	1067	5	9.9 (0.09)
NW_ATPew _(h = 2) vs RW (h = 1)	1067	6	8.5 (0.08)
RW (h = 1) vs NW_CR2coyI+CR3coyII _(h = 15)	1067	1	3.5 (0.03)
NW_ATP2coyI _(h = 10) vs NW_ATP3coyII _(h = 6)	1067	0	5.4 (0.05)
OW_ATP4gwNA _(h = 3) vs OW_ATP4gwEU _(h = 5)	1067	1	6.5 (0.06)

Calculations were done in DnaSP v. 5.10. Gaps were excluded from the analysis. Comparative groups are identified in Figures 2a and 2b, and RW is the red wolf sequence identified with superscript "ruf" in Figures 2a and 2b. The dog sequence was excluded from analyses and Ccr19 was excluded from the OW_CR5gwEU _grouping because it clustered independently from North American and Eurasian lineages (see Figure 2a). NW = New World; OW = Old World; CR = control region.

Based on the control region, divergence of eastern wolves from coyotes was approximately 3.0%, that of the red wolf sequence from coyotes was 2.0%, and eastern wolves compared to red wolves was 2.7% (Figure 3). These values are consistent with those reported for a 238 bp control region fragment (3.2%, 2.3%, 2.1%, respectively) [19]. As expected, divergence estimates from the ATPase region were lower (0.9%, 0.3%, and 0.8%, respectively) but were proportionally consistent with that from the control region. These results complement the phylogenetic analysis and show further confirmation for the eastern wolf lineage. Differences between OW evolved North American and Eurasian wolves were 1.8% for the control region and 0.6% at the ATPase region, suggesting a closer relationship between these lineages than between NW lineages.

TMRCA estimates from both genetic regions were similar and suggest divergence of eastern wolf sequences from other North American evolved sequences at approximately 486,300 - 548,400 years ago (ya), and North American grey wolves from Eurasian wolves at 465,600 - 518,100 ya, although the 95% highest posterior densities (HPD) had wide intervals (Table 3). These values for NW divergence are higher than previous TMRCA estimates of 150,000 - 300,000 ya [19] but results are not entirely inconsistent when HPD is considered. Our estimate of NW coalescence is closer to that proposed for coyotes of 420,000 ya in [18], although that study did not identify distinct eastern wolf haplotypes because it occurred prior to identification of eastern wolf sequences [19].

**Table 3:** TMRCA estimates

Diverged Lineages	Clades	Mean TMRCA (mya)	SEM	Geometric mean	95% HPD
**CR**					
Eastern wolf-Coyote	NW_CR1ew_-NW_CR2coyI+CR3coyII _	0.4863	0.0054051	0.3302	0.00091379 - 1.224
NA grey wolf-EU wolf	OW_CR4gwNA_-OW_CR5gwEU _	0.5181	0.0035853	0.3624	0.0042139 - 1.2689
OW-NW	OW_CRall_-NW_CRall_	1.3961	0.0018707	1.3704	0.8906 - 1.9061
**ATPase**					
Eastern wolf-Coyote	NW_ATP1ew_-NW_ATP2coyI+ATP3coyII _	0.5484	0.0016439	0.4889	0.139 - 1.0834
NA grey wolf-EU wolf	OW_ATP4gwNA_-OW_ATP4gwEU _	0.4656	0.0014502	0.4124	0.1153 - 0.9494
OW-NW	OW_ATPall_-NW_ATPall_	1.8343	0.0012901	1.8224	1.4352 - 2.2465

Estimates based on Bayesian analysis of mtDNA control region (CR) and ATPase region in BEAST. Eastern wolf is represented by sample CAN004377 from Algonquin Provincial Park in Ontario, Canada; Coyote is represented by sample CAN000142 from Texas, United States; North American grey wolf is represented by CAN001806 from the Northwest Territories, Canada, Eurasian wolf is represented by a grey wolf from Russia (Genbank Accession DQ480503). Clades are identified in Figures 2a and 2b. OW = Old World; NW = New World.

### Selection

Tests of neutrality showed significance in NW lineages, particularly in the ATPase6 region; for ATPase8, only Fu's *Fs *identified departure from neutrality (Table 4). Over the past 100 years, grey wolves have experienced a genetic bottleneck [37], whereas coyote populations have expanded across North America [23,27], presenting the two extremes of demographic history. Significantly negative values of neutrality statistics can be indicative of selection but are also consistent with either population subdivision or expansion [38], and Fu's *Fs *is a particularly powerful test of population growth [39,40]. Therefore, it is difficult to disentangle selection from demographic history when interpreting neutrality tests [1,41] and care should be taken not to over interpret results from a rejection of the neutrality hypothesis. The known demographic history, however, suggests that results from neutrality tests presented here are indicative of the population expansion of coyotes.

**Table 4:** Tests of neutrality within clades and regions

Clade or Region	Sample size	Tajima's *D*	Fu & Li D*	Fu & Li F*	Fu's *Fs*
**ATPase6**					
All	25	-0.20630 (P > 0.10)	-1.05880 (P > 0.10)	-0.92649 (P > 0.10)	-6.0687 (P < 0.05)**
NW_ATPall_	18	-2.23648 (P < 0.01)**	-2.90725 (P < 0.02)**	-3.14575 (P < 0.02)**	-13.0395 (P < 0.001)**
NW_ATP2coyI+ATP3coyII_	16	-2.24950 (P < 0.01)**	-3.10889 (P < 0.02)**	-3.30776 (P < 0.02)**	-11.6113 (P < 0.001)**
NW_ATP2coyI_	10	-1.94429 (P < 0.05)**	-2.27595 (P < 0.02)**	-2.46830 (P < 0.02)**	-4.6976 (P < 0.01)**
NW_ATP3coyII_	6	-1.11000 P > 0.10)	-1.19719 (P > 0.10)	-1.27471 (P > 0.10)	-2.6587 (P < 0.05)**
OW_ATPall_	7	-0.65997 (P > 0.10)	-0.71815 (P > 0.10)	-0.77484 (P > 0.10)	-1.2687 (P > 0.10)
OW_ATP4north_	4	-0.388921 (P > 0.10)	-0.38921 (P > 0.10)	-0.37908 (P > 0.10)	-0.9463 (P > 0.10)
OW_ATP4gwEU_	5	-0.38168 (P > 0.10)	-0.38168 (P > 0.10)	-0.40360 (P > 0.10)	0.4897 (P > 0.50)
**ATPase8**					
All	25	0.06944 (P > 0.10)	-0.42777 (P > 0.10)	-0.32087 (P > 0.10)	-1.5442 (P > 0.20)
NW_ATPall_	18	-1.66626 (0.10 > P > 0.05)	-1.79890 (P > 0.10)	-2.03413 (P > 0.10)	-6.4462 (P < 0.001)**
NW_ATP2coyI+ATP3coyII_	16	-1.63027 (0.10 > P > 0.05)	-1.97065 (0.10 > P > 0.05)	-2.15788 (0.10 > P > 0.05)	-5.5160 (P < 0.01)**
NW_ATP2coyI_	10	-1.74110 (P < 0.05)**	-2.01007 (0.10 > P > 0.05)	-2.17902 (0.10 > P > 0.05)	-2.2598 (P < 0.02)**
NW_ATP3coyII_	6	-0.05722 (P > 0.10)	0.07132 (P > 0.10)	0.04654 (P > 0.10)	-2.4288 (P < 0.02)**
OW_ATP4all_	7	-0.65405 (P > 0.10)	-0.51900(P > 0.10)	-0.59207 (P > 0.10)	0.1098 (P > 0.40)
OW_ATP4gwNorth_	4	n/a	n/a	n/a	n/a
OW_ATP4gwEU_	5	-0.17474 (P > 0.10)	-0.17474 (P > 0.10)	-0.17531 (P > 0.10)	0.0607 (P > 0.30)

Sample size is the number of sequences analyzed based on haplotypes determined from the complete ATPase sequence (1067 bp). Dog sequence Catp04 was excluded from analysis because it clustered independently and may have been influenced by breeding selection. OW_ATP4North _includes haplotypes Catp03 and Catp18 from the NWT, Canada, Catp08 from Sweden, and Catp09 from Russia; n/a indicates test not applicable because there were no polymorphic sites. Significantly negative values are indicative of selection, population subdivision or expansion. ** indicates statistical significance.

The high rate of synonomous substitutions (SS) in the ATPase genes, particularly in the ATPase6 region where *d*N/*d*S ratios were all < 0.3, indicate that purifying selection has been influential in *Canis *mtDNA evolution, particularly in NW lineages (Table 5). This excess of synonomous substitutions in mtDNA coding regions is consistent with that previously found in wolves, coyotes and dogs [42] and in other mammals including humans [17,43] and mice [11]. This pattern is thought to mainly affect terminal branches of phylogenetic trees suggesting recently diverged groups show a stronger synonomous substitution signal [16], which is consistent with the pattern observed in our dataset.

**Table 5:** Comparison of nonsynonomous (NSS) and synonomous (SS) substitutions in ATPase6 and ATPAse8 genes among clades

Clade	Sample Size	NSS	SS	*d*N/*d*S
**ATPase6**				
All	25	11	52	0.045
NW_ATPall_	18	5	23	0.073
NW_ATP1ew_	2	1	4	0.082
NW_ATP2coyI+ATP3coyII_	16	5	17	0.088
NW_ATP2coyI_	10	2	9	0.073
NW_ATP3coyII_	6	3	8	0.112
OW_ATP4all_	7	4	9	0.163
OW_ATP4North_	4	3	4	0.282
**ATPase8**				
All	25	10	13	0.171
NW_ATPall_	18	4	6	0.295
NW_ATP1ew_	2	0	0	0.000
NW_ATP2coyI+ATP3coyII_	16	3	6	0.214
NW_ATP2coyI_	10	1	4	0.070
NW_ATP3coyII_	6	2	2	0.285
OW_ATP4all_	7	3	0	n/a
OW_ATP4North_	4	0	0	0.000

Sample size is the number of sequences analyzed based on haplotypes determined from the complete ATPase sequence (1067 bp). Dog sequence Catp04 was excluded from the analysis because it clustered independently from other Old World (OW) sequences. OWNorth includes haplotypes Catp03 and Catp18 from the NWT, Canada, Catp08 from Sweden, and Catp09 from Russia. The ratio of nonsynonomous to synonomous substitutions is calculated as *d*N/*d*S, where *d*N is the number of nonsynonomous substitutions per nonsynonomous site, and *d*S is the number of synonomous substitutions per synonomous site. All calculations were done in DNAsp v. 5.10. Clades are those identified in Figure 2b.

The ratio of nonsynonomous to synonomous substitutions (*d*N/*d*S ) was highest for OW lineages from northern climates in the ATPase6 region, but the same pattern was not observed for ATPase8 (Table 5). The difference in *d*N/*d*S ratios between OW and NW lineages is not unusual given that mutations can be neutral in some lineages but non-neutral in others [44]. It has been proposed that amino acid variation in the ATPase genes may reduce the efficiency of oxidative phosphorylation, thereby decreasing ATP production while increasing heat production thus conferring a selective advantage for certain haplotypes in colder climates [13-15], although this hypothesis is not without controversy [8,16,17]. The higher *d*N/*d*S ratios for ATPase6 in OW evolved lineages in northern climates compared to NW lineages from more temperate and subtropical climates (Table 5) hints at a possible role for adaptive selection in response to climate for *Canis *mtDNA evolution [13], but statistical tests of nonsynonomous and synonomous substitutions did not support adaptive selection in northern regions (Table 6). Although a wolf poisoning campaign in the NWT during the 1950s [45] may have decreased genetic diversity of NWT grey wolves somewhat, it is unlikely to have impacted haplotype diversity to the extent that targeted extermination efforts did in the US [37]. Further complicating interpretation is the recent suggestion that analysis of *d*N/*d*S ratios and the McDonald-Kreitman test are ineffective at detecting positive selection [46,47].

**Table 6:** P values for lineage and climatic comparisons of nonsynonomous and synonomous substitutions

Comparison	Fisher's Exact Test	McDonald-Kreitman Test
**ATPase6**		
NW_ATPall _vs OW_ATP4all_	0.4288	0.3017
NW_ATPall _vs OW_ATP4North_	0.3117	0.287
**ATPase8**		
NW_ATPall _vs OW_ATP4all_	0.1923	0.4015
NW_ATPall _vs OW_ATP4North_	n/a	n/a

Fisher's exact test is based on raw numbers of nonsynonomous substitutions (NSS) and synonomous substitutions (SS); McDonald-Kreitman test is based on comparisons *d*N/*d*S for fixed differences between populations and polymorphism within a population. n/a indicates the test was not applicable because there were no substitutions observed for OW_North _at ATPase8. Dog sequence Catp04 was excluded from analysis because it clustered independently and may have been influenced by breeding selection. OW_North _includes haplotypes Catp03 and Catp18 from the NWT, Canada, Catp08 from Sweden, and Catp09 from Russia. Clades are those identified in Figure 2b. Higher *d*N/*d*S values in samples from more northern climates suggest a selective advantage at colder temperatures.

The discrepancies in our analysis and in the human literature does not necessarily mean that climatic adaptation has not influenced the evolution of mtDNA lineages, but suggests rather that mtDNA evolution is more complex than climatic variation alone can explain [8]. Although further investigation on a larger dataset with an alternative approach [15] may help clarify the role of climate in shaping the distribution of *Canis *mtDNA, and could provide valuable insight into the observed patterns of mtDNA introgression in eastern North American populations, it will remain difficult to separate a signal for adaptive selection from the dramatic and contrasting demographic histories of *Canis *populations. The development of novel analytical tools will be required to adequately disentangle natural selection from demographic processes.

## Conclusions

Here, we provide important new data for phylogenetic inference of wolves and coyotes in North America. We know of no other study that reports as extensively on the ATPase region in wild *Canis *species. Similar patterns of diversity and divergence between the control region and ATPase regions suggest that evolutionary patterns can be inferred from non-coding regions of mtDNA. Overall phylogenetic concordance between the control and ATPase regions suggests that the control region can be an informative marker for inferring gene trees when dealing with recent divergence. Of particular importance is the monophyletic clustering of eastern wolf sequences under a new Bayesian analytical approach, thereby providing further evidence for a distinct North American evolved wolf, independent of coyotes and grey wolves, that inhabited the temperate forests of eastern North America prior to colonization by European settlers. In addition, eastern wolf sequences are further diverged from coyotes than the red wolf sequence. This does not necessarily imply that the red wolf is not a distinct species but rather supports the assignment of coyote-like sequences as eastern wolf specific.

Understanding the role that selection has had on mtDNA evolution and distribution is a more difficult task. Although the high rate of synonomous substitutions provides evidence that purifying selection may have influenced the evolution of *Canis *mtDNA, especially in NW lineages, the role of adaptive selection in response to climate is more ambiguous. Adaptive selection may play a role in the geographic distribution of OW mtDNA sequences in North America, but alternative analytical approaches will no doubt be required to adequately test this hypothesis.

Based on the human literature, however, it seems probable that climate is influential in adaptive selection of mtDNA. Further research on adaptive selection of *Canis *mtDNA is particularly important because it provides a mechanism by which eastern wolf and coyote like mtDNA have introgressed extensively into grey-eastern wolf hybrids in northern Ontario and the Great Lakes region [24,26,27,32,48] and why eastern wolf mtDNA is prevalent in eastern coyote populations [23,27,33,34]. It is important to note that mtDNA introgression can occur with little or no obvious nuclear introgression [49-51], and in some cases completely replace mtDNA in the absence of apparent nuclear introgression [50,52]. It is possible, therefore, that introgression of NW mtDNA lineages into grey-eastern wolf hybrids in eastern North America, and introgression of eastern wolf mtDNA into eastern coyotes, reflect chance or rare events on which selection then acted creating species discordance between mtDNA and the nuclear genome [1]. Overall, this research provides a new and important framework with which to study patterns of mtDNA introgression and geographic distribution in species where taxonomy has been blurred by incomplete lineage sorting and/or hybridization.

## Methods

### Sequencing

Previously extracted DNA from 83 individuals from various North American *Canis *species (eastern wolves, grey wolves, red wolves, and coyotes) were selected to represent a broad spectrum of geographic regions and mtDNA haplotypes. Extraction methods are provided elsewhere [19,53]. Nine additional sequences analyzed in [42] were obtained from GenBank (Accession Numbers: DQ480499, DQ480503 - DQ480508, DQ480510, and DQ480511). Locations for samples used in the present analysis are listed in Additional File 1. All polymerase chain reaction (PCR) amplifications were conducted in a 20 μL reaction under the following conditions: approximately 1ng DNA, 1.5 mM MgCl_2_, 0.2 mM dNTPs, 0.2 μM forward primer, 0.2 μM reverse primer, 0.1 μg bovine serum albumin (BSA), 1 × PCR buffer, and 1 Unit of *Taq *DNA polymerase (Invitrogen, Burlington, Ontario). PCR cycles were run with an initial denaturation at 94°C for 5 minutes, followed by 30 cycles of 94°C for 30 seconds, 56°C, 58°C or 60°C (depending on the region being amplified) annealing for 30 seconds, and 72°C extension for 30 seconds with a final extension at 72°C for 4 minutes. A ~425 bp fragment of the mtDNA control region was amplified with primers ThrL: 5'-GAA TTC CCC GGT CTT GTA AAC C-3' and DLH-can: 5'-CCT GAG GTA AGA ACC AGA TG-3' [22] under a 60°C annealing temperature. Three primer pairs described in [42] were used to amplify a region that contained the ATPase6 and ATPase8 region of the mtDNA (For7651: 5'-CTT TAT ACC CAT TGT TCT TG-3' and Rev8248: 5'-GGC GTA AAT GAG TGA GGT AAT-3' (597 bp; 56°C annealing); For8049: 5'-CCA TTT TAT TCC CAA CAC CC-3' and Rev8501: 5'-GGT AGC CCC TCC ATT CAA A-3' (452 bp; 58°C annealing); For8255: 5'-CAA CTC TCT ATA AAC CTC GG-3' and Rev8891: 5'-CGT ATC GTA GTC CTT TTT GTA-3' (636 bp; 58°C annealing). We designed additional primer pairs for two of the regions because variation in the primer sites in coyotes led to inefficient amplification. For7651B: 5'-CTT TAT GCC CAT TGT TCT TG-3' and Rev8248B: 5'-GGT GTA AAT GAG TGG GGT AAT-3'; For8049B: 5'-CCA TTT TAT TCC CAG CAC CC-3' and Rev8501B: 5'-GGT AGC CCC TCC AAT CAA G-3'. PCR products were cleaned with ExoSAP-IT (USB Corporation, Ohio) and analyzed on either a MegaBace 1000 (GE Healthcare Bio-Sciences, Baie d'Urfé, Quebec) or an AB 3730 (Applied Biosystems Canada, Streetsville, ON). Contigs from forward and reverse sequences were assembled in Sequencher 4.9 (GeneCodes Corporation, Michigan) and edits were made based on visual inspection of electropherograms. Of the 83 samples, 74 full control region sequences of 347 bp (72 had both forward and reverse, 2 from one direction only) and 80 ATPase region sequences of 1067 bp were generated (base pairs 7729 - 8795 in the *Canis *mtDNA complete genome sequence DQ480510; Region 1: 70 samples in both forward and reverse directions, 10 in one direction only; Region 2: 77 in both forward and reverse directions, 3 in one direction only; Region 3: 79 in both forward and reverse directions, 1 in one direction only). These, plus the 9 sequences from GENBANK, were used in subsequent analyses. Sequence alignment was done with ClustalW implemented in Geneious 4.5 for Mac OSX (Biomatters Ltd., NZ, http://www.geneious.com) followed by visual inspection.

### Diversity and phylogenetic analysis

Measures of DNA sequence variation within and among groups, including number of haplotypes (h), variable sites, nucleotide diversity per site (Pi), and average number of nucleotide differences between groups were calculated for the control region, the full ATPase sequence, ATPase6, and ATPase8 in the software program DnaSP v5.10 [54]. Calculations for ATPase6 and ATPase8 were limited to a subset of groups analyzed with the full 1067 bp sequence because the number of variable sites was lower in the specific gene regions, especially in ATPase8, due to the shorter fragment size. Phylogenetic analysis was conducted under a Bayesian framework implemented in the program BEAST v. 1.4.8 [55]. We combined 3 independent runs, each with 10,000,000 MCMC iterations while sampling from the chain every 1000 steps. We used a relaxed uncorrelated lognormal molecular clock [56] with a substitution rate of 3.8 × 10^-8^/year for the control region [37,48] and 1.5 × 10^-9 ^for the ATPase region based on the median rate for substitution at the cytochrome b region in carnivores [57]. Based on Bayesian Information Criteria (BIC) generated in ModelGenerator [58] we used an HKY [59] with invariant sites model with a transition/transversion rate ratio kappa of 45.04 and a 0.79 fraction of invariable sites. As we were interested in putative species phylogenies, we used a Yule tree prior as recommended in the BEAST manual. For the ATPase region, we used an HKY model with a gamma distribution and 4 rate categories, alpha of 0.21 and a transition/transversion rate ratio kappa of 42.96. To ensure high performance and accuracy, the BEAST output was scrutinized in the software TRACER v. 1.4.1 [60]. Raw traces for all parameters suggested the MCMC had converged on a stationary distribution, and all effective sample size (ESS) values were over 300 (most were over 1000). A burnin of 10% was used when annotating output files. Maximum clade credibility trees were visualized in FigTree v.1.2.3 http://tree.bio.ed.ac.uk/software/figtree/.

### Divergence and TMRCA

Divergence between clades was estimated by comparing the number of fixed differences, the average number of nucleotide differences, and the average number of nucleotide substitutions per site with a Jukes and Cantor correction (D_xy_JC_) [61] calculated in DnaSP v5.10 [54]. We also compared divergence of the putative red wolf sequence with the eastern wolf clade and other sequences in the broader coyote clade.

Time to most recent common ancestor (TMRCA) was estimated in BEAST software with parameters described above. Four sequences were selected at random to represent the main clades: DQ480503 (Ccr15; Catp09) represented the OW Eurasian lineage, CAN001806 (Ccr03; Catp03) represented the OW North American lineage, CAN004377 (Ccr12; Catp16) represented the eastern wolf lineage, and CAN000142 (Ccr26; Catp22) represented the coyote lineage. Tree calibration was done by setting the divergence distribution between OW and NW lineages based on fossil evidence at 1.5 million years ago and a standard deviation of 0.5 million years such that the 95% range would be 1 - 2 mya [19,62].

### Selection

For all the haplotypes identified in the ATPase phylogenetic tree (n = 25, dog excluded) we examined the ATPase6 (681 bp) and ATPase8 (204 bp) regions to test for selection. To test the hypothesis of neutral evolution, we used DnaSP v 5.10 [54] to calculate Tajima's *D *[63], Fu and Li's D*, Fu and Li's F* [64], and Fu's *Fs *[65] overall and within clades. Significance values for each test are based on the confidence limits of *D*, the critical values of D* and F* [64], or with 1000 replicates in the coalescent simulations approach of DnaSP v. 5.10 for Fu's *Fs*. We also tested for neutrality in a group of OW sequences from northern climates (Russia, Sweden, and Northwest Territories, Canada).

To determine whether purifying or adaptive selection influenced the evolution of *Canis *mtDNA, we compared the number of nonsynonomous substitutions (NSS) to synonomous substitutions (SS), and the ratio of nonsynonomous substitutions per nonsynonomous site to synonomous substitutions per synonomous site (*d*N/*d*S) in ATPase6 and ATPase8 overall, within clades, and in the group of OW sequences from northern climates. To test whether climate may have influenced *Canis *mtDNA distribution, we compared *d*N/*d*S at ATPase6 and ATPase8 for all NW sequences compared to a) all OW sequences and b) compared to OW sequences from northern climates. If climate were a factor in the adaptive selection of mtDNA, one would expect higher *d*N/*d*S ratios in northern climates compared to more temperate regions [13]. Significance of differences was determined with a two-tailed Fisher's exact test on the raw data (NSS and SS) with the online calculator available at http://faculty.vassar.edu/lowry/VassarStats.html (accessed October 22, 2009) and a McDonald-Kreitman test [66] conducted in DnaSP v. 5.10.

## Abbreviations

ATPase: adenosine triphosphatase; Catp: *Canis *adenosine triphosphatase; Ccr: *Canis *control region; COX: cytochrome oxidase; coy: coyote (*Canis latrans*); CR: control region; EU: Eurasia; EW: eastern wolf (*Canis lycaon*); fam: dog (*Canis lupus familiaris*); HPD: highest posterior density; mtDNA: mitochondrial DNA; mya: millions of years ago; NA: North America; NW: New World; NWT: Northwest Territories; OW: Old World; ruf: red wolf (*Canis rufus*); RW: red wolf (*Canis rufus*); SEM: standard error of the mean; TMRCA: time to most recent common ancestor.

## Authors' contributions

LYR conducted all the laboratory work and molecular genetic analyses, and drafted the manuscript. BRP provided samples, was involved in the research study concept, and helped revise the manuscript. BNW guided the laboratory work and molecular genetic analyses, was involved in the research study design, and revised the manuscript. All authors contributed intellectual input as well as read and approved the final manuscript.

## Supplementary Material

Additional file 1 **Sample information and haplotype summary**. *Canis *mtDNA control region and ATPase haplotype summary with comparisons of control region haplotypes found in this study to previously published literature. Click here for file
